# Amniotic-Fluid Stem Cells: Growth Dynamics and Differentiation Potential after a CD-117-Based Selection Procedure

**DOI:** 10.4061/2011/715341

**Published:** 2011-02-23

**Authors:** S. Arnhold, S. Glüer, K. Hartmann, O. Raabe, K. Addicks, S. Wenisch, M. Hoopmann

**Affiliations:** ^1^Department of Veterinary Anatomy, Justus-Liebig-University Giessen, Frankfurter Straße 98, 35392 Giessen, Germany; ^2^Department of Anatomy I, University of Cologne, 50924 Cologne, Germany; ^3^Clinic for Obstetrics and Gynaecology, University of Tuebingen, 72016 Tuebingen, Germany

## Abstract

Amniotic fluid (AF) has become an interesting source of fetal stem cells. However, AF contains heterogeneous and multiple, partially differentiated cell types. After isolation from the amniotic fluid, cells were characterized regarding their morphology and growth dynamics. They were sorted by magnetic associated cell sorting using the surface marker CD 117. In order to show stem cell characteristics such as pluripotency and to evaluate a possible therapeutic application of these cells, AF fluid-derived stem cells were differentiated along the adipogenic, osteogenic, and chondrogenic as well as the neuronal lineage under hypoxic conditions. Our findings reveal that magnetic associated cell sorting (MACS) does not markedly influence growth characteristics as demonstrated by the generation doubling time. There was, however, an effect regarding an altered adipogenic, osteogenic, and chondrogenic differentiation capacity in the selected cell fraction. In contrast, in the unselected cell population neuronal differentiation is enhanced.

## 1. Introduction

Fetal stem cells represent a relatively new cell population in the field of stem cell research, exhibiting unique and fascinating features, while recent studies have provided new insights into stem cell biology as well as new putative strategies to exploit their therapeutic potential. These cells can be derived from the fetus itself or alternatively from supportive extraembryonic structures such as the amniotic fluid [1]. The combination of being extraembryonic and providing relatively easy access for cell harvesting makes the amniotic fluid an important alternative source of fetal stem cells [2, 3].

It is, however, well known that amniotic fluid represents a very heterogeneous population that includes cells derived from the fetal membranes as well as from the fetus itself. Amniotic fluid- derived cells have been classified on the basis of their morphological, biochemical and growth characteristics, with three groups of amniotic-fluid-derived cells identified such as the epithelial-like cells, amniotic fluid-specific cells, and fibroblast like cells [4]. 

At the beginning of the amniotic fluid-derived cell culture out of the amniotic-fluid-specific cells preferentially epithelial-like cells can be found, while fibroblast like cells usually appear later and not in all fluid samples. These cells are thought to be derived from the fibrous connective tissue and dermal fibroblasts [4, 5].

The development of specific culture protocols for AF-cells has permitted the isolation and expansion of multipotent stem cells from the amniotic fluid 6. Thus, these stem cells exhibit typical mesenchymal stem cell markers such as CD 29, CD 90, CD 166, CD 73, CD 105, CD 49, and CD 117 2, and they are negative for the hemopoietic surface markers CD 45, CD 34, and CD 14 [6, 7]. Further investigations revealed that these cells also have characteristics of multipotent stem cells, as demonstrated by the expression of stem cell markers such as Oct-4 and SSEA-4 [2, 3]. Apart from the differentiation potential along the mesodermal lineage by differentiation into osteoblasts and chondrocytes also the differentiation into the neural lineage has been documented [6]. Further investigation using CD 117 positive AF-cells have confirmed the potential of these cells to differentiate toward neural lineages [2]. In regard of their pluripotency they are an attractive autologous cell source for fetal tissue engineering applications. So far, autologous amniotic fluid cell-based tissue engineered constructs have been successfully used for neonatal repair of the diaphragm and for fetal trachea reconstruction [8], and they have the potential to form fetal heart valve leaflets in vitro [9]. An enhanced neural marker expression of immortalized amniotic fluid cells has been shown after application of neural induction [10]. 

Therefore, in our study we have investigated the growth characteristics of AF cells in different culture conditions obtained from ordinary amniocenteses as well as from amniodrainages. The focus of our study was the differentiation potential of AF cells after a cell sorting procedure using the MACS system on the basis of the surface marker CD 117. Our data reveal that AF cells from the positive fraction show improved differentiation characteristics into the osteogenic and chondrogenic lineage, while cells from the negative fraction, favourably show a neural differentiation capacity. After intracerebral transplantation in adult rats, cells show a remarkable integration and differentiation capacity.

## 2. Material and Methods

### 2.1. Origin of Amniotic Fluid-Derived Cells

Amniotic fluid was obtained from human amniocentesis samples. Amniocentesis is a clinical routine procedure performed during week 15–20 of gestation for prenatal diagnosis. It is an ethically approved and accepted procedure. Alternatively, amniotic fluid was taken from amniodrainages from patients with polyhydramnios. Cell samples were only used when no major abnormalities were revealed by the cytogenetic analysis. All patients gave written informed consent and were informed in detail about our procedures for diagnostic fulfillment according to the protocol of this study, which was approved by the local hospital ethics committee.

### 2.2. Cell Culture of Amniotic Fluid-Derived Cells

The amniotic fluid-derived cells were routinely cultured in Minimum Essential Medium (*α*-MEM; Invitrogen, Karlsruhe, Germany) supplemented with 10% fetal bovine serum (FBS, PAA, Cölbe, Germany) in a 100 mm culture dish and incubated at 37°C with 5% humidified CO_2_. Alternatively cell culture was carried out in advanced Dulbeccos Modified Eagles medium (ADMEM, Invitrogen) supplemented with 5% FBS. The first medium change was performed after 5 days in order to avoid any mechanical stress. After 10 to 14 days, when the cultures had reached 80% confluence, cells were harvested using Accutase (PAA), and were replated at 2000 cells/cm^2^. The cells were kept in culture for up to 6 passages. For the experiments cells were taken from passage 2.

Alternatively to standard culture conditions, amniotic fluid-derived cells were cultured in an atmosphere with a reduced O_2_ tension. Normal humidified tissue culture incubators with 5% CO_2_ were used for the 21% oxygen cultures. For decreased oxygen cultures, plates were cultivated in the presence of 2% O_2._

### 2.3. Evaluation of Cell Kinetics

Cell kinetics were studied by determining the generation doubling time of cells from amniodrainages and amniocenteses as well as from selected and unselected AF cells. Cells were plated in culture medium at a density of 1 × 10^3^ cells/cm^2^ in cell culture dishes and allowed to multiply until 80% confluence. Adherent AF cells were then lifted and counted with a haemocytometer at every passage. Generation doubling times (GDT) and numbers (CD) were calculated from hemocytometer counts and cell culture time (CT) for each passage according to the following 2 formulae:

(1) $$\text{CD}=\frac{\ln\;\left(N1/N2\right)}{\ln\;\left(2\right)},\;\;\text{GDT}=\frac{\text{CT}}{\text{CD}}$$

(*N*1: number of cells at confluence prior to passaging and *N*2: number of cells seeded after passaging).

### 2.4. Construction of HC-Ad Vectors and Cell Labeling

The high-capacity adenoviral vector (HC-Ad) was generated as previously described [11]. The vector HC-Ad.FK7 expressing enhanced GFP (EGFP-enhanced green fluorescent protein) has also been described [11]. AF cells were infected using a stock AdV concentration of 2 × 10^6^ infectious units/*μ*L, corresponding to 50 multiplicities of infection (MOI) for a single cell. Transduction efficiency was analysed 24 h after transduction by determining the population of cells with EGFP expression within the total cell population labeled by the Hoechst dye nuclear stain (not shown).

### 2.5. Immunoselection for CD117

For immunoselection of c-Kit-positive cells from single-cell suspensions, the cells were incubated with a rabbit polyclonal antibody to CD117 (c-Kit), specific for the protein's extracellular domain (amino acids 23–322) (Santa Cruz Biotechnology, Santa Cruz, CA, USA).

The CD117-positive cells were purified by incubation with magnetic Goat Anti-Rabbit IgG MicroBeads and selection on a Mini-MACS apparatus (Miltenyi Biotec, Bergisch-Gladbach, Germany) following the protocol recommended by the manufacturer. AF cells were subcultured routinely at a dilution of 1 : 4 to 1 : 8 and not permitted to expand beyond 70% of confluence.

### 2.6. CFU Assay

Following MACS sorting and expansion CD117+ and CD117− amniotic fluid-derived cells were plated at 10,50, 100, 500, 1.000, and 10.000 cells/100 mm diameter culture dish. After12 days of culture, cells were fixed and stained with 1% cresyl violet in 100% methanol. The number of colonies with >50 cells was counted.

### 2.7. AF-Cell Differentiation In Vitro

In vitro differentiation was carried out as described previously for mesenchymal stem cells [12]. Cells were analyzed for their capacity to differentiate into the osteogenic and chondrogenic lineage. Differentiation was performed in a monolayer. Cells were plated at a density of 3 × 10^3^ cells/cm^2^, and cultured at 37°C in a humidified atmosphere (5% CO_2_) for various periods. The medium was changed three times a week.

Osteogenic differentiation was induced by culturing AF cells for 3 weeks in osteogenic medium (OM). OM consisted of DMEM (Invitrogen) containing 10% FCS, 0.05 mM ascorbic acid-2-phosphate, 10 mM *β*-glycerophosphate, and 0.1 *μ*M dexamethasone. Osteogenic differentiation of ASCs was assessed by evaluating morphological changes and calcified extracellular matrix (ECM) deposition. 

Induction of chondrogenic differentiation was carried out in a three-dimensional (3D) pellet culture. For 3D pellet cultures AF cells were resuspended at a density of 5 × 10^5^ cells/mL. The chondrogenic medium was supplemented with insulin-transferrin selenium (ITS) 1 : 100 v/v (1.0 mg/mL insulin from bovine pancreas, 0.55 mg/mL human transferrin, and 0.5 *μ*g/mL sodium selenite), 0.1 *μ*M dexamethasone, 0.05 mM ascorbic acid, 50 *μ*M l-proline, 1 mM sodium pyruvate (Sigma, Deisenhofen, Germany). Cell suspension (500 *μ*L) was aliquoted into 15 mL polypropylene centrifuge tubes (Eppendorf, Hamburg, Germany), and centrifugated at 500 g for 5 min. as described previously [12]. The chondrogenic medium was supplemented with 5 ng/mL of TGF-*β*1 (Sigma). Fresh medium was added every third day. The medium without TGF-*β*1 was used as the control medium for control 3D culture. The tubes were placed in the incubator at 37°C in a humidified atmosphere with 5% CO_2_ for up to 3 weeks. The caps of the tubes were loosened to allow air exchange.

### 2.8. Cell Type Examination

For histological staining procedures, cells that were cultivated according to the osteogenic or chondrogenic induction procedure were fixed with 4% PFA, dehydrated, embedded in paraffin and cut into 5 *μ*m sections. The cells were stained with alkaline phosphatase, von Kossa or the Alcian blue staining in order to demonstrate the osteogenic and chondrogenic differentiation potential, respectively. For the von Kossa staining the cells were fixed with 4% formaldehyde for 30 min at room temperature. The cells were rinsed with distilled water and then overlaid with a 1% (wt/vol) silver nitrate solution in the absence of light for 30 min. The cells were washed several times with distilled water and were counterstained with nuclear fast red.

### 2.9. Neural Induction of Amniotic Fluid-Derived Cells

For the enhancement of neural differentiation, amniocyte-derived cells were transferred to a serum-free medium (DMEM/F12, Invitrogen) supplemented with the growth and differentiation factor FGF8 (100 ng/mL) in combination with retinoic acid (RA) 1 *μ*M and plated at a density of 2000 cells/cm^2^ on Poly-Ornithine-Laminine coated glass coverslips in 4-well culture plates. Alternatively, they were cultivated in DMEM/Hams F12 supplemented with 10% FBS, epidermal growth factor (EGF, 20 ng/mL, R&D systems, Wiesbaden, Germany), basic fibroblast growth factor (bFGF, 20 ng/mL), and N2 (Invitrogen). Cells were cultivated for 5 days in these induction media prior to processing for immunocytochemical investigations.

### 2.10. Transplantation of EGFP-Labelled AFC ells

For intraventricular injection, adult Wistar rats (250–300 g) were used and were kept in accordance with Federal Government guidelines. Animals were anesthetized with a mixture of 6 mg/kg xylazine and 60 mg/kg ketamine. The animals were transferred to a stereotactic apparatus. An incision was made in the scalp 3 mm lateral to the bregma. A burr hole was made 0.3 mm caudal and 2.5 mm lateral to the bregma using a dental drill. 10 *μ*L of predifferentiated and GFP labeled AF-cells were slowly injected into the striatum at a depth of 4.5 mm from the dura [13]. Following human AF-cell injection the animals received daily subcutaneous cyclosporin A (Sandoz, Switzerland) injections (10 mg/kg). After 7, 14, and 21 days, rats were sacrificed by intracardiac perfusion under deep anesthesia with xylazine and ketamine in PBS supplemented with heparin and procainhydrochloride, followed by 4% formaldehyde. The brains were removed, the forebrains trimmed, and the samples frozen after cryoprotection in 18% sucrose. Cryostat sections (20 *μ*m) were processed for identification of GFP-expressing grafted cells and for immunofluorescence analysis.

### 2.11. Immunocytochemical Staining of Cells

For the investigation of neural marker expression by amniotic fluid-derived cells, the cells of 5 independent cultures were fixed after rinsing in 0.1 M PBS in 4% PFA. Cryostat sections of the brain were processed for immunohistochemical analysis as well. Cells and tissue sections were treated for 30 min. with a blocking solution composed of 2% goat serum and 5% FBS. In experiments requiring the labelling of intracellular epitopes, the cells and tissue sections were further treated with 0.025% Triton X-100 for 30 min and rinsed in PBS. 

The primary antibodies used in this study are presented in Table 1. 

Incubations were performed over night at 4°C. Secondary antibodies were used at the following dilutions: Cy3 or Cy2-conjugated affinity-purified goat anti-mouse or anti-rabbit IgG (Rockland, Gilbertsville, PA, USA) 1 : 1000.

Anti-GFAP antiserum produced in guinea pig was detected by Biotin-conjugated goat antiguinea pig (Sigma, Germany) at a dilution of 1 : 400 followed by FluoroLinkTMCyTM3 labelled streptavidin (Amersham Pharmacia Biotech, Freiburg, Germany) at a dilution of 1 : 1000. Incubations were performed for 1h at room temperature. After antibody incubations nuclear DNA was stained with Hoechst Dye 33342. Preparations were coverslipped with Entellan (Merck, Darmstadt, Germany). Analysis of mounted specimens was carried out using an Axiophot Fluorescence microscope (Zeiss, Oberkochen, Germany).

Negative controls were carried out by omitting the primary antibodies. Specificity of neural markers was tested using appropriate cell populations such as neural stem cells and neural stem-cell-derived neurons and glial cells.

### 2.12. Statistical Analysis

The cells were viewed using an Axiophot Fluorescence microscope (Zeiss, Oberkochen, Germany). Cells in five consecutive random fields were counted for each coverslip. The number of cells was expressed as mean ± SD from at least two-to-four coverslips from three independent experiments. Statistical analysis, including the evaluation of the ELISA investigations, was carried out Applying the Students *t*-test or alternatively the one way analysis of variance (ANOVA) using the Microcal Origin software (Northampton, MA, USA).

## 3. Results

### 3.1. In Vitro Characterisation of Amniotic Fluid-Derived Cells

AF cells were allowed to adhere to plastic culture dishes and were grown for 4-5 passages in *α*-Modified Eagles Medium (*α*-MEM) supplemented with 10% fetal bovine serum (FBS) or alternatively in an Advanced Dulbeccos Modified Eagles Medium (ADMEM) supplemented with 5% FBS. In both media the first adherent cells could be identified 24 hours after plating. Morphological examination revealed that cells do not form a monolayer of cells adherent to the bottom of the culture dish. Instead several dense cell clusters can be observed. After the first passage though, a homogenous cell layer (monolayer) can be seen, however, the resulting cell population is rather heterogeneous. Growing in islands or in cell groups, cells revealed polygonal morphologies establishing close contacts to the neighbouring cells. Besides, there were also elongated spindle shaped cells, resembling mesenchymal cells in vitro. 

**Figure 1:**
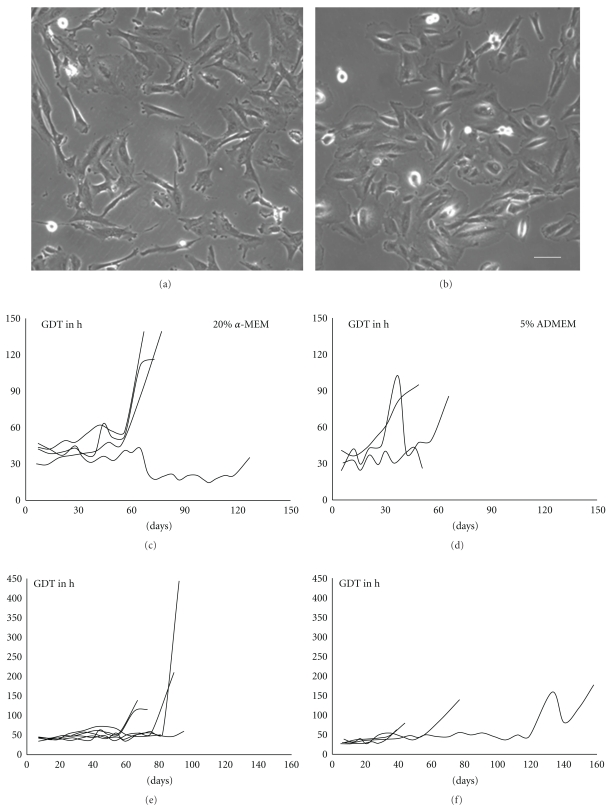
Morphology and growth characteristics of AF cells under different culture conditions. (a) AF cells cultivated in the presence of *α*-MEM supplemented with 10% FBS exhibit an elongated morphology. (b) In the presence of ADMEM with 5% FBS cells revealed a rather epithelioid shape. (c) Cells cultivated in *α*-MEM (10% FBS) showed a more homogenous generation doubling time (GDT) (d) compared with those cultivated in ADMEM (5% FBS) (*n* = 5). (e) The GDT of cells derived from amniocenteses is rather homogenous with an average survival time of 60–80 days. (f) Cells derived from amniodrainages reveal somewhat longer survival times than cells derived from amniocenteses and their growth dynamics are inhomogenous (*n* = 5). Scale bar in a and b = 25 *μ*m.

After the first passage AF cells show marked morphological differences dependent on the culture medium used. All together the heterogeneity of the cell population was still apparent, but while cells cultivated in *α*-MEM with 10% FBS were mainly spindle shaped with elongated processes, cells cultivated in ADMEM with 5% FBS had a morphology resembling epithelial cells in vitro (Figures 1(a) and 1(b)).

The proliferation capacity of AF cells was compared along with the cultivation in the two different culture media. In both media the generation doubling time could be determined as 30 to 40 hours. While in the medium (*α*-MEM) supplemented with 10% FBS cells revealed a stable proliferation rate until day 60 in passage 10, while with cells cultivated in ADMEM supplemented with 5% FBS a stable growth could only be observed until day 30 or the 4th to 5th passage (Figures 1(c) and 1(d)). Because of these more stable culture conditions for further experiments only cells cultivated in *α*-MEM (10% FBS) were used.

As a next step cell growth of cells derived from ordinary amniocentesis was compared with that of cells obtained from amniodrainages. As the volume of the amniotic fluid from the amniodrainages was 1000-fold higher than that from amniocenteses, consequently the cell yield of amniodrainages was also markedly higher. Morphologically, in culture dishes from amniodrainages huge amounts of desquamated epithelial cells could be found. However, despite of the markedly higher original cell number in amniodrainage-derived preparations the cell density of about 700–1200 cells/cm^2^ was similar between the two cell populations. Comparing the long-term culture of both cell populations it was evident that the growth characteristics of cells derived from amniocenteses is more constant with an average survival time of 60 to 80 days. Consequently, for our further experiments only those cells were used (Figures 1(e) and 1(f)).

### 3.2. Magnetic Bead-Associated Cell Separation

**Figure 2:**
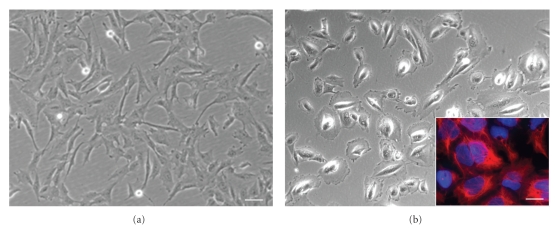
Morphological analysis of CD117+ and CD117– AF cells. (a) CD117+ cells reveal the typical elongated morphology of mesenchymal stem cells, (b) CD117− AF cells display a rather polymorph appearance of epithelial cells in vitro, inset, CD117− cells can be stained by the epithelial marker cytokeratin (red fluorescence) in a counterstain with the nuclear marker Hoechst dye (blue), scale bar in a and b = 25 *μ*m, and in the inset in b = 15 *μ*m.

An immunoselection procedure with magnetic microspheres (MACS) was carried out in order to isolate the c-Kit–(CD 117) positive stem cell population from the heterogeneous amniotic fluid-derived specimens as CD 117 is considered to be specific for mesenchymal stem cells [2]. After the selection procedure the percentage of CD117+ cells was 3.2 ± 1.03% of the total cell population. Furthermore, after cell separation morphologically the CD117+ cell fraction revealed the typical features of mesenchymal stem cells with elongated cell bodies (Figure 2(a)), while the CD117− cell fraction consisted of cells revealing a polymorph morphology resembling epithelial cells in vitro (Figure 2(b)), using immunocytochemistry these latter cells could be shown to express cytokeratin typical for epithelial cells (inset in Figure 2(b)). The generation doubling time was evaluated in unselected cells as well as in the CD 117 positive and the CD 117 negative cell fraction. Data from the growth dynamics reveal that the generation doubling time with an average of 30–40 hours is not altered after the selection procedure. It remains constant until day 60 in culture (not shown).

### 3.3. CFU Assay

**Figure 3:**
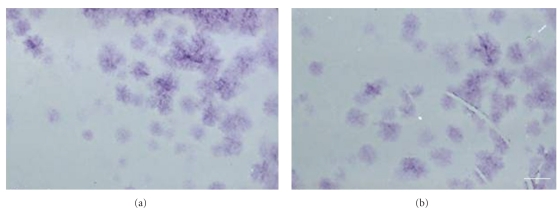
CFU assay for the demonstration and comparison of the self-renewing potential of CD117− and CD117+ AF-cells. Plating of very low cell numbers leads to the generation of single colonies (CFU) as demonstrated by the cresyl violet staining. (a) Colony formation after plating of 1000 CD117(−) cells per 100 mm culture dish. (b) Colony formation after plating of 1000 CD117(+) cells per dish. Scale bar = 1 cm.

The potential of self-renewal was analyzed by use of the CFU assay. Cells were plated at a density of 10, 50, 500, 1.000, 5.000, and 10.000 cells/100-mm culture dish and were expanded for 12 days. Individual colonies were generated even when AF cells were plated at low densities such as 5 and 10 cells/culture dish. The mean rate was 30% of the starting AF-cell population, indicating a subpopulation of cells with self-renewing capacity in vitro. There were no differences between CD117+ and CD117− cells (Figure 3).

### 3.4. In Vitro Differentiation Potential of AF Cells

To investigate multipotentiality of amniotic fluid derived cells, AF-cells were cultivated in specific induction media for the characterisation of the osteogenic, chondrogenic and neuronal differentiation potential in vitro.

**Figure 4:**
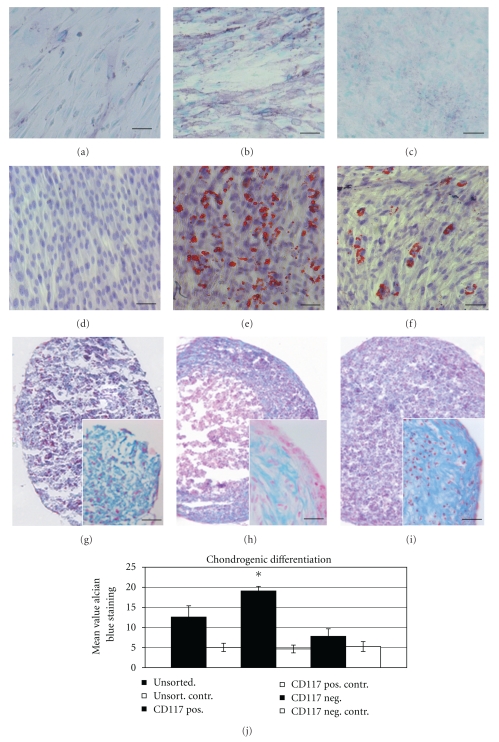
In vitro differentiation potential of AF cells into the osteogenic (ALP staining), adipogenic, and chondrogenic (Alcian blue staining) lineage. (a) while there was only a weak ALP staining in the CD117− cell fraction, (b) this was markedly stronger in the CD117+ cell population. (c) Unselected AF-cells also reveal a rather weak osteogenic differentiation as demonstrated by a weak ALP staining. (d) While no adipogenic differentiation as demonstrated by the Oil RedO staining could be observed in the CD117– cell fraction, (e) strong positive Oil Red O staining could be observed in the CD117+ fraction, (f) in the unselected population only a minor number of cells showed a Red Oil O positive staining. (g) After chondrogenic induction in CD 117 negative cells there is a diffuse Alcian blue staining detectable. (h) After chondrogenic induction the CD 117 positive cell fraction forms a firm pellet with a strong Alcian blue staining in the cortical zone. The pellets have a hyaline cartilage-like morphology based on characteristic lacunae formation containing round to oval shaped cells which resemble chondrocytes, which occur singly or in nests of two to three cells. (i) Diffuse Alcian blue staining can also be detected in unselected cells. (j) Densitometric analysis of Alcian blue staining in the different experimental groups There is a significant higher staining intensity in the CD117+ cell fraction compared to unselected or CD117− cells (*n* = 6). Scale bar in (a–i) = 100 *μ*m, in the insets in (g–i) = 50 *μ*m.

The osteogenic potential of the cell population was tested after a cultivation period of 21 days under conditions which were suitable to induce the differentiation of MSCs into the osteogenic lineage 12. In the presence of ascorbate, dexamethasone, and *β*-glycerophosphate, cells proliferated rapidly and formed densely packed colonies. In culture, dense granular areas appeared within individual colonies and over the time multiple layers of cells often formed in these cultures. Osteogenic differentiation was evaluated in unselected AF cells as well as in the CD117+ and the CD117− cell fraction. There was a markedly stronger ALP staining intensity in the CD117+ cell population than in the CD117− or in the unselected cell fraction (*n* = 5) (Figures 4(a)–4(c)). 

Similar to the ALP activity, a continuous increase in calcium deposition as analysed by the von Kossa staining could be demonstrated (not shown). Adipogenic differentiation was similarly evaluated using the Red Oil O staining (*n* = 5). While in CD117− cells there is no adipogenic differentiation detectable, this is most prominent in the CD117+ cell fraction. Unselected cells reveal a moderate adipogenic differentiation capacity (Figures 4(d)–4(f)).

Chondrogenic differentiation was induced using a three-dimensional pellet culture [14] (*n* = 5). Under these conditions three-dimensional cell aggregates could be detected already two days after chondrogenic induction. 

The aggregates gradually increased in size over the total cultivation period of three weeks. After that time the aggregates were fixed, cryoprotected, and embedded in Tissue Tec and frozen at −80°C. Cryosections of the pellets were stained using the Alcian Blue staining specific for proteoglycans (Figures 4(g)–4(i)). According to the osteogenic differentiation also with the chondrogenic differentiation there is a markedly stronger Alcian blue staining intensity in the CD117+ cell fraction compared to the unselected and CD117− cell population. In case of the CD117+ cell population this is significantly stronger (*P* < .05) than in appropriate control cells cultivated without chondrogenic differentiation factors (Figure 4(j)).

In order to further prove the differentiation potential of AF cells and to establish their possible application in cell-based therapies for neurodegenerative diseases also their neuronal differentiation capacity was evaluated in the CD117+ as well as in CD117− and unselected cells. 

**Figure 5:**
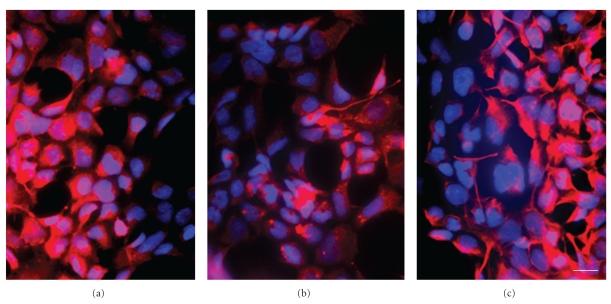
Neural marker expression as a proof for the neural differentiation potential. After the neural selection procedure the expression of the neural markers such as (a) *α*-internexin, (b) *β*III tubulin, (c) as well as GFAP can be shown. Secondary antibodies for all primary antibodies are Cy2 conjugated (red fluorescence). Cell nuclei are labelled with the Hoechst dye 33342nuclear stain (blue fluorescence). Scale bar in (a–c) = 10 *μ*m.

**Figure 6:**
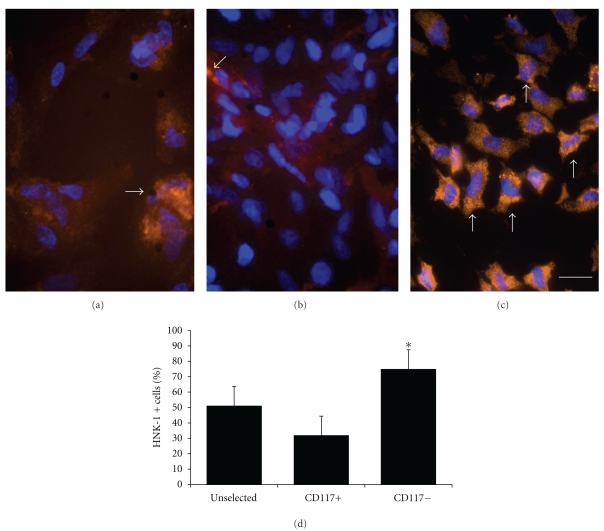
HNK-1 expression in AF cells as a confirmation of neuronal differentiation capacity in the CD 117 positive and negative cell fraction and under hypoxic (2% O_2_) culture conditions. (a) Moderate HNK-1 (orange fluorescence, arrow) expression in unselected cells and (b) a weak expression of the early neuronal marker HNK-1 (arrow). (c) While there is a stronger HNK-1 expression in CD 117 negative cell fraction detectable (arrows), (d) graphical display about the quantification of HNK-1 expression in the unselected, CD117+ and CD117− cell fractions. ^(∗)^significant increase of HNK-1 immunopositive cells compared to control. Secondary antibodies are Cy2 conjugated (red to orange fluorescence. The whole cell population is detected with the Hoechst dye nuclear stain (blue fluorescence) (*n* = 5, *P* < .05). Scale bar in (a–f) = 25 *μ*m.

Neuronal differentiation was carried out in the presence of neuronal induction factors as well as under hypoxic conditions (2% pO_2_). The neuronal differentiation potential of CD117− and stimulated cells was analysed using specific neural markers such as *β*III tubulin, alpha internexin, and GFAP (Figure 5). Furthermore, the increase in neuronal differentiation after neuronal induction was quantified compared to unselected cells as well as CD117+ cells by determination of the expression of the early neuronal marker HNK-1 [15]. Evaluation of the HNK-1 expression revealed that there is a significantly stronger expression of this marker in the negative cell fraction than in the unselected as well as in the CD117+ cell population (Figure 6).

### 3.5. Neuronal Characteristics of AF Cells after CNS Transplantation

**Figure 7:**
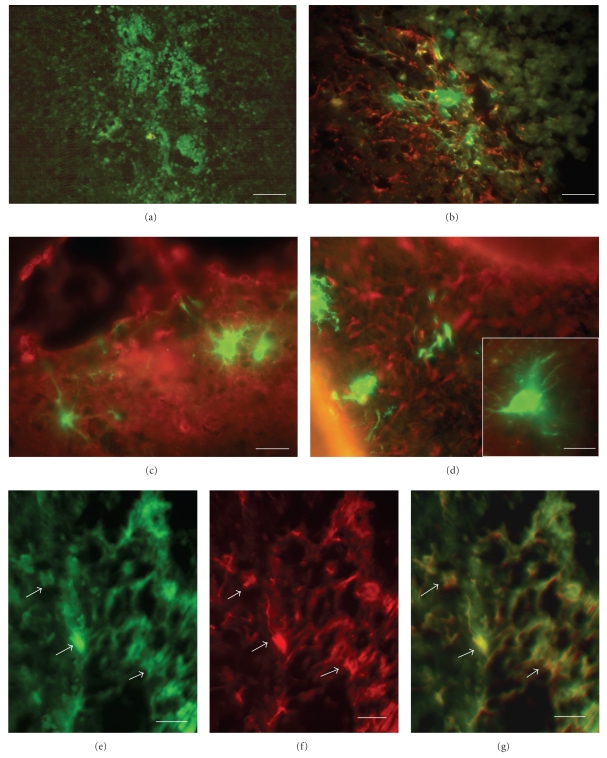
Integration and differentiation of GFP labelled (green fluorescence) predifferentiated CD117− AF cells after grafting into the striatum of adult rats. (a) GFP labelled cells can be found around the injection site in the striatum. (b) one week after grafting GFP expressing cells are still arranged in clusters near the injection site, in a co staining with the astrocyte marker GFAP (Cy2 conjugated secondary antibody, red fluorescence) (c) and (d) typical neural cell morphologies of GFP expressing cells with elongated processes can be detected 2 weeks after grafting in a costaining with the early neuronal marker *α*-internexin (Cy2,red), (e) a dense network of GFP expressing processes are visible, (f) which show a co-staining with the astrocyte marker GFAP (Cy2, red). (g) Overlay of GFP (green) and GFAP (red) expressing cells. Scale bar in (a) = 20 *μ*m, in (b) = 15 *μ*m and in (c) and (d) = 10 *μ*m.

GFP-expressing AF cells cultured in selection medium were injected stereotactically into the striatum of adult Han Wistar rats. One week after injection, cells could be easily identified by their GFP expression in histological sections of the injection site (Figure 7(a)). At that time cells revealed a round and uniform morphology without any processes, which is typical for undifferentiated cells [16]. Over a period of 1–3 weeks following grafting, cells could be detected at various locations. Cells had the typical morphology of neural cells with neurite-like processes. At 2 weeks after grafting, the majority of EGFP-expressing cells were arranged in cell clusters and are partially coexpressing GFAP (Figure 7(b)); at 3 weeks after grafting a dissemination of cells was visible. Morphological investigations revealed typical neuronal morphologies with prominent processes (Figures 7(c) and 7(d)). Immunofluorescence analysis using an antibody against GFAP revealed a morphological integration of EGFP-labeled AF cells into the environment of host cells within the striatum, preferentially in the subventricular space with a clear colabeling of GFP and GFAP (Figures 7(e)–7(g)).

## 4. Discussion

It is demonstrated that culture conditions and derivation of amniotic fluid cells are decisive for the cultivation and expansion of certain subtypes of heterogenous AF derived cells. 

According to reports in the literature, a high serum content (20% FBS) in conjunction with the alpha MEM medium promotes a mesenchymal phenotype of cells [6]. In our investigation these cells reveal rather constant growth characteristics, while the use of ADMEM with a serum content as low as 5% results in the enrichment of a more epithelial cell population without any uniform growth dynamics. Furthermore, in order to obtain a more homogenous cell population, the method of amniotic fluid retrieval is rather important. If the fluid is obtained from amniodrainages, the cellular yield of intact mesenchymal cells is rather low. Instead, there is a large amount of desquamated epithelial cells which disturbs the adherence of intact cells. The adherent cells also reveal rather inhomogeneous growth dynamics. To our knowledge this is the first comparison of cells derived from either amniodrainages in order to relieve a polyhydramnios or from the amniotic fluid under normal conditions from patients during pregnancy between the 15th and the 20th week of gestation. 

In order to obtain a more homogenous cell population and to isolate mesenchymal stem cells, one option is a surface marker associated cell sorting as realized by the MACS technology. However, the surface markers observed for AF cells are not consistent among reports from different groups. While some laboratories have found that AF cells are negative for CD117 [6, 17], another group was able to isolate AF cells with CD117-bounded magnetic microspheres [2]. However, the expression levels of other surface markers such as CD105 have also been found to differ among research groups. Some groups have found that CD105 is strongly expressed in AF cells [2, 17], while another group reported that CD105 is only weakly expressed [6]. Because there are so many different reports about the expression profile of AF cells, there is the need for an indepth investigation to further characterize these cells. Our results indicate that only about 2–5% of the total cell population was positive for CD117. However, after cell separation the CD117+ cell fraction revealed the typical features of mesenchymal stem cells, while the negative population has a rather epithelial morphology. In contrast, surprisingly all three cell populations: the unselected cells, the CD 117 positive cell fraction as well as the CD 117 negative cells show similar growth characteristics. This indicates that although the cell population within the amniotic fluid is heterogenous, a coexistence of various cell populations is possible.

The differentiation potential of CD 117 positive and negative cells, respectively, was studied by their adipogenic, osteogenic, chondrogenic as well as their neuronal differentiation potential. Unsurprisingly, the CD 117 positive cell fractions revealed markedly better adipogenic, osteogenic as well as chondrogenic differentiation characteristics compared to the negative or unselected AF cells. These data again show the mesenchymal properties of the selected cell population and are thus in accordance to previous reports about the broadly pluripotent quality of AF cells [14, 18].

In contrast the neuronal differentiation potential, as demonstrated by the expression of the early neuronal marker HNK-1, is higher in the CD 117 negative cell fraction than in the CD 117 positive cell population. This is in sharp contrast to the report by De Coppi et al. [2], who have shown that the CD 117 positive cells show a neuronal differentiation potential. 

This discrepancy may partially be caused by the great heterogeneity of the original cell population as well as by the culture conditions, including the serum batch used. On the other hand as already described earlier there are studies applying flow cytometry, which reveal that amniotic fluid cells are negative for CD117 [6]. In fact, this may actually indicate that there are two different stem cell populations present in the amniotic fluid, one which is CD117+ and another one which is CD117−. The latter would be the one in our study with an improved neuronal differentiation potential. Anyway, as both CD117+ and CD117− amniotic fluid cells isolated in our study show a self-renewing property and show a multidifferentiation potency, the quality and the methodological approach including the isolation procedure are comparable and as reliable as those described in the current literature. 

Neuronal characteristics of amnion fluid-derived cells have also been described earlier by our and other groups [10, 19]. Neuronal differentiation in our study was carried out under hypoxic culture conditions as it is known that hypoxia stimulates neuroregeneration in the ischemic brain [20]. 

The feature of neuronal properties and neuronal differentiation characteristics can also be confirmed by our transplantation studies. After GFP labelling in order to unequivocally detect amniotic fluid- (AF-) derived cells, integration into various parts of the central nervous compartment can be demonstrated. Cells reveal the typical characteristics of neural cells with a multitude of processes. Additionally, cells also coexpress several neuronal and glial markers such as GFAP indicating their integration and also their in situ differentiation along the neural lineage. These data are in line with earlier reports about the integrative capacity of AF cells in the rat brain [21] and our own observations concerning the neuronal differentiation potential of bone marrow-derived mesenchymal stem cells and their integration properties after transplantation into the adult rat brain [13].

## 5. Conclusion

Altogether, our data reveal the multipotentiality of stem cells from the amniotic fluid, which emphasizes that amniotic fluid cells could be of interest as an alternative source of stem cells for the treatment of various diseases. However, dependent on the desired cell differentiation or therapeutic application the separation procedure has to be adapted accordingly. While mesenchymal cells rather differentiate into the osteogenic, adipogenic, or chondrogenic lineage, the remaining cell population including epithelial cells can be favourably directed into the neural lineage. Especially, in neonatal medicine stem cells from the amniotic fluid can be applied as an autologous cell source for diseases that have to be treated directly after birth such as osteogenic or chondrogenic defects as well as neurodegenerative diseases including the retinopathy of prematurity.

##  Funding

The work was in part funded by the Elke-Kröner-Fresenius-Stiftung, Bad-Homburg, Germany.

## Figures and Tables

**Table 1:** 

Antibody	Source	Dilution
Anti-CD 117, rabbit polyclonal	Santa Cruz Biotechnology, Santa Cruz, CA, USA	1 : 11
Anti-pan Cytokeratin, mAb	Abcam, Cambridge, UK	1 : 40
Anti-*α*-internexin mAb	Chemicon, Temecula, CA, USA	1 : 1000
Anti-GFAP mAb	Sigma, Deisenhofen, Germany	1 : 400
Anti-*β*-III-tubulin, antiserum produced in rabbit	Covance, Richmond, CA, USA	1 : 8000
Anti-HNK-1 mAb, hybridoma supernatant, TIB 200	American Type Culture Collection, Manassas VA, USA	1 : 10
